# EXPOSURE TO GENERATIVE MESSAGES BOOSTS COGNITIVE PERFORMANCE IN OLDER ADULTS

**DOI:** 10.1093/geroni/igac059.2466

**Published:** 2022-12-20

**Authors:** Tara Gruenewald, Natalie Standridge, Clarissa Tadros

**Affiliations:** Chapman University, Orange, California, United States; Chapman University, Orange, California, United States; Chapman University, Orange, California, United States

## Abstract

Both self-perceived and primed generativity has been found to be linked to better cognitive and physical functioning, psychological well-being, and longevity in older adulthood. A prior experiment also found that priming older individuals with messages regarding the generative, or contributory value of their social group (older adults) boosted cognitive performance compared to exposure to a message about the societal burden of one’s group (Hagood & Gruenewald, 2018) . A key limitation of this earlier work was the lack of a neutral prime condition to determine whether being exposed to messages regarding the generativity of older adults enhanced their cognitive performance or being exposed to negative messages regarding the social burdens of one’s age group suppressed performance. This limitation was addressed in the current online experiment of 300 U.S. adults age 55 and older who were randomly exposed to either a social generativity, social burden, or neutral prime presented as a test of reading comprehension and recall. Participants completed other measures of cognitive ability before and after the priming task, including an assessment of verbal memory. An ANCOVA model including age as a covariate indicated a significant effect of priming condition (F(2,296) = 3.30, p = .038). Mean verbal memory performance did not vary between the neutral and social burden priming conditions, while performance was significantly higher in the generativity prime condition (d=.29). Experimental findings provide support for the hypothesis that exposure to generative messages about one’s social group can boost cognitive performance in a national sample of older adults.

